# Behavioral Convergence with Physiological Divergence: Sex Differences in Hormones but Not Social Behavior in Beagle Dogs

**DOI:** 10.3390/ani16111680

**Published:** 2026-05-30

**Authors:** Yu-Huan Xiao, Zi-Hua Zhao, Xue-Yan Jiang, Jun Zhang, Wen-Bing He, Rui Dong, Xue-Ting Zhang, Li-Xian Tao, Jun-Lv Ma, Jin-Xiu Li, Ya-Ping Zhang

**Affiliations:** 1 State Key Laboratory of Genetic Evolution & Animal Models, Kunming Institute of Zoology, Chinese Academy of Sciences, Kunming 650201, China; 2 Kunming College of Life Science, University of Chinese Academy of Sciences, Kunming 650201, China; 3 Bio-X Center for Interdisciplinary Innovation, Yunnan University, Kunming 650500, China; 4 School of Life Science, Yunnan University, Kunming 650500, China; 5 School of Ecology and Environmental Science, Yunnan University, Kunming 650500, China

**Keywords:** Beagle dogs, Social interactions, experimenter gender effect, hormones, domestication

## Abstract

The “experimenter gender effect” is a pervasive confound in rodent behavioral neuroscience: the sex of the human handler alters stress, social, and pharmacological responses via olfactory cues and conserved neural circuits. Whether this effect extends to dog—a species co-domesticated with humans for over 15,000 years—has never been systematically tested. Here, we examined sex-biased social preferences in Beagle dogs during both intra- and cross-species interactions, and asked whether baseline neuroendocrine states predict such preferences. Thirty-four adult Beagles (17 males, 17 females) from a standardized laboratory colony underwent social interaction tests with same and opposite-sex conspecifics and with male and female experimenters. Baseline plasma corticosterone, serotonin (5-HT), and dopamine were measured by ELISA. Results indicated that Beagles did not exhibit significant sex-based preferences for either gender of conspecifics or human experimenters in either dog–dog or human–dog social interaction tests (all *p* > 0.05). However, males showed markedly higher baseline corticosterone, 5-HT, and dopamine than females (all *p* < 0.0001), a hormonal dimorphism that did not correlate with any behavioral measure in Spearman correlation analysis (*p* > 0.05). Nevertheless, this study has several limitations: only baseline hormone levels were measured (not stress-induced responses), behavioral tests involved only low-stakes affiliative interactions, and only one breed was studied under standardized conditions. These results suggest that Beagle dogs may lack experimenter-gender preference in social interactions, exhibiting stable, gender-neutral social behavior despite profound underlying hormonal differences. This decoupling of internal state from behavioral output suggests that domestication may have shaped a social phenotype resistant to the experimenter gender effect, supporting the Beagle as a valuable translational model with a stable baseline and low susceptibility to confounding social cues, making it suitable for research on affective and social-cognitive disorders.

## Simple Summary

This study investigates whether Beagle dogs exhibit gender preferences in social interactions, both with other dogs and with human experimenters. Using standardized behavioral tests, we found that neither male nor female Beagles showed significant preference for interacting with either gender of conspecifics or humans. Additionally, plasma hormone analysis revealed that male dogs had significantly higher levels of corticosterone, serotonin (5-HT), and dopamine compared to females. Despite these neuroendocrine differences, no gender-based behavioral preferences were observed. These results suggest that Beagle dogs may serve as a less biased behavioral model for neuropsychiatric research, reducing the confounding influence of experimenter gender often observed in rodent models.

## 1. Introduction

Rodents, particularly rats and mice, are the most widely utilized animal models in psychology and behavioral neuroscience. Rodent behavioral analysis constitutes a primary specialized domain within experimental psychology and behavioral neuroscience, as rodents exhibit a broad repertoire of species-typical behaviors under controlled laboratory conditions [1]. Global initiatives to consider sex as a biological variable emerged amid technological advances in behavioral neuroscience. However, the field’s historical over-reliance on male rodents has created male-biased experimental paradigms, leading to potential misinterpretations when studying females. Consequently, it is now common to find experimental effects present in males but not in females [2]. Current research indicates that, despite the NIH requiring funded researchers to consider sex as a biological variable (SABV) and include subjects of both sexes in all experiments, male rodents remain the default model. This persistent bias may lead to misdiagnoses and an increased risk of adverse drug reactions [3]. The sex of the experimenter critically influences rodent performance across behavioral paradigms, affecting anxiety-like behaviors, social interactions, and cognitive function. Its manifestation and magnitude is modulated by the animal’s sex, strain, and experimental context [2,3,4]. In rodent models, the differential impact of experimenter sex on anxiety-like behaviors has been extensively documented. Compared to female experimenters, the presence or residual odor of male experimenters can induce stronger stress and anxiety-like responses in mice. For instance, in tests such as the elevated plus maze, mice exhibit reduced exploratory activity in the presence of male experimenters. Mice showed aversion to the scent of male experimenters, preference for the scent of female experimenters and increased stress susceptibility when handled by male experimenters [5]. In unconditioned threat-based anxiety tests, such as the elevated plus maze and open field test, the anxiety-like behavior of female animals is significantly influenced by the stage of the estrous cycle, with anxiety levels increasing during specific phases [6]. Therefore, experimenter sex has emerged as a critical confounding factor that may interfere with the accurate assessment of anxiety, social behavior, and cognitive function, while also challenging the reliability and cross-laboratory reproducibility of experimental results.

Rodents identify chemical cues associated with the experimenter’s sex via the olfactory system. Studies have demonstrated that the odor of male experimenters can induce an aversion response in female rats. Female rats exhibited consistently exacerbated anxiety-related behaviors along with elevated body surface temperature during repeated exposure to male experimenters. This was accompanied by increased circulating corticosterone levels and decreased oxytocin. Notably, similar physiological responses could be replicated using T-shirts worn by human males, confirming the critical role of olfactory cues [7]. These studies demonstrate that the sex of the human experimenter affects not only anxiety and pain responses [8], but also depression-relevant behaviors in mice. The findings suggest that the sex of human experimenters may more broadly be considered a factor that can influence rodent behavior and experiments that measure behavioral outcomes [3,9]. Ketamine is an effective rapid-acting antidepressant at subanesthetic doses clinically; the sex of the experimenter significantly modulates the pharmacological effects of ketamine. Ketamine reduced forced swim test immobility time only when given by male experimenters; no such effect occurred with female experimenters. Likewise, male experimenter administration uniquely increased hippocampal synaptic GluA1 levels and enhanced high-frequency EEG oscillations (30–120 Hz). These findings suggest that environmental stressors, such as the scent of the experimenter, may serve as a critical permissive factor for ketamine’s rapid antidepressant effects. This observation may also explain the difficulty in replicating robust ketamine efficacy in some laboratory settings, particularly those with predominantly female experimenters or lower baseline environmental stress. Furthermore, these effects are predominantly mediated by olfactory signals: human male scent selectively activates corticotropin-releasing factor (CRF)-expressing neurons in the entorhinal cortex, which project to hippocampal CA1—a neural circuit implicated in the differential behavioral responses observed under male versus female experimenter conditions [9,10,11]. Earlier findings of Sorge et al. demonstrated that exposure to the scent of male experimenters resulted in higher corticosterone levels, stress-induced analgesia and increased anxiety-like behaviors in mice [8].

Mammalian societies exhibit remarkable diversity, which must be understood through a multi-dimensional framework encompassing social structure, caregiving systems, and mating systems. Within this framework, rodents, canids, and humans differ fundamentally in their social organization and behavioral strategies—differences that may profoundly influence their susceptibility to experimenter-derived social cues such as gender. In rodents, social hierarchies are established through aggression and resource competition; social interaction in rodents enables the transmission of food preferences, novel environment exploration, and danger information [12,13]. Humans are regulated by social hierarchy and cultural and institutional factors. From an early age, they rely on the social environment to learn survival skills. The transmission of social information serves as the foundation for the continuation of culture [14]. In canids, human-like social skills have evolved, and behavioral synchronization acts as an interspecific social glue between dogs and humans [15]. These profound differences in social system organization and behavioral repertoires between rodents and canids (including dogs) offer a compelling explanation for why the “experimenter gender effect” is so robust in rodents but may be attenuated or absent in domestic dogs. Rodents’ reliance on olfactory-driven, resource-dependent social networks makes them highly sensitive to human-derived chemical cues. Whereas dogs’ evolutionary history as descendants of pack-living wolves with cooperative breeding systems, and their adaptation to human companionship, has favored a social orientation more attuned to multimodal (visual, auditory) signals from humans—a shift that likely mitigates, but does not eliminate, the influence of experimenter sex as a behavioral modulator in specific contexts. This perspective aligns with studies showing that human–dog relationships share features of attachment bonds, with dogs using their owners as a secure base and safe haven during exploration and distress [16,17], and that empathy, attachment, and anthropomorphism are key psychological mechanisms shaping the quality of human–animal relationships [18]. Therefore, the dog—and specifically the well-standardized Beagle model, if it provides a unique opportunity to examine social behavior that may be largely free from the confounding influence of experimenter gender—will offer a more stable and translationally relevant baseline for neuropsychiatric research.

The pervasive “experimenter sex effect” in rodent models has raised serious concerns regarding internal validity and cross-laboratory reproducibility, as the sex of the experimenter—a social variable that is difficult to fully standardize—can profoundly interfere with the interpretation of behavioral phenotypes and pharmacological responses. To some extent, this limits the internal validity and translational reliability of these models for studying complex psychiatric disorders, particularly those involving social cognition and affective interaction [4,6,9,19,20]. Consequently, the search for alternative animal models with more stable behavioral baselines and reduced susceptibility to experimenter-derived social cues has become a priority in translational neuroscience. The domestic dog (Canis familiaris) has emerged as a compelling candidate in this regard. Through tens of thousands of years of coevolution with humans, dogs have developed sophisticated socio-cognitive abilities, advanced emotional recognition, and neuroanatomical pathways that bear notable similarities to humans. Dogs have rapidly evolved from a traditional pharmacological model into a cutting-edge platform for research on various neuropsychiatric disorders [21,22,23,24]. Beagles, in particular, are widely used in preclinical research due to their genetic homogeneity, manageable temperament, and high reproductive efficiency, and have been successfully deployed in studies of traumatic brain injury, cognitive aging, and neurodevelopmental disorders [25,26,27]. Most notably, CRISPR/Cas9-engineered Shank3 mutant Beagles recapitulate core autism-like phenotypes, including social withdrawal and elevated anxiety, demonstrating the breed’s utility for modeling complex behavioral dysfunction [28,29]. Studies have found that in healthy dogs aged 1–7 years across nine breeds, serum 5-HT levels were significantly higher in females than in males, with breed differences also being observed [30]. However, another study involving different dog breeds reported that plasma levels of 5-HT, cortisol, and norepinephrine were significantly lower in females than in males. Regardless of sex or paw preference, stroking the animals alleviated stress responses, increased 5-HT levels, and decreased cortisol levels, suggesting that environmental interactions modulate neurotransmitter activity [31]. It should be noted that both corticosterone and cortisol can serve as indicators of stress hormones in dogs. Furthermore, research has found that dogs diagnosed with Attention-Deficit/Hyperactivity Disorder (ADHD) exhibit lower serum levels of 5-HT and dopamine, which are significantly correlated with aggression, hyperactivity, impulsivity, fear, and tactile sensitivity [32]. Corticosterone and cortisol levels in dogs are influenced by housing environment and human interaction, making them key indicators for assessing animal welfare and stress status. However, relatively few reports are currently available on sex differences in corticosterone, 5-HT, and dopamine in dogs.

Although canine behavioral research has expanded considerably in recent years, existing studies on sex-related social preferences in dogs remain limited and yield conflicting conclusions. Some reports, primarily involving shelter dogs, have observed modest anxiety-related behaviors or heightened aggression scores toward unfamiliar men compared to unfamiliar women during behavioral tests or leash walks [26,33]. However, these findings are often confounded by heterogeneous rearing conditions, diverse breed backgrounds, and unpredictable early-life social experiences, which limit their generalizability to laboratory-reared Beagle dogs maintained under standardized conditions. Furthermore, social behavior in dogs operates across two distinct domains: interactions with conspecifics (dog–dog) and interactions with humans (dog–human). These domains likely engage partially overlapping but distinct cognitive and neural systems, with intraspecific behavior rooted in canid evolutionary history and interspecific behavior shaped by thousands of years of domestication selecting for cooperative, non-aggressive interactions with humans [34,35]. Notably, domestication has altered dogs’ conspecific social organization compared to their closest non-domesticated relatives, gray wolves [36]. Wolves live in packs whose survival depends on coordinated behavior, whereas dogs rely less on conspecifics—predicting greater group cohesion in wolves than in dogs. Endocrine correlates such as oxytocin and glucocorticoids modulate group cohesion, resulting in species-specific differences in social interactions. Importantly, although wolves and dogs showed similar observable behavioral reactions to a territorial threat and separation from the pack, their hormonal responses differed. Only wolves exhibited positive correlations between oxytocin/glucocorticoid concentrations and territorial behaviors, and only wolves showed increased glucocorticoid concentrations after separation [36]. This dissociation between behavior and endocrine state underscores that behavioral measures alone may not fully capture underlying physiological processes. Critically, no study has simultaneously examined Beagle dogs intra- and cross-species social sex preferences within a unified experimental framework, nor integrated behavioral measures with baseline neuroendocrine profiling.

Given these considerations, a fundamental question remains unresolved as the Beagle model gains prominence in translational neuroscience: do Beagle dogs themselves exhibit systematic sex differences or preferences in social interactions? To address this knowledge gap, the present study employs a unified experimental framework to systematically characterize social behavior in healthy male and female Beagle dogs. We quantified behavioral responses in standardized same- and opposite-sex interactions with both conspecifics and human experimenters, and further integrated baseline plasma neuroendocrine profiling (corticosterone, 5-HT, dopamine) to explore potential physiological correlates of social behavior. Our primary objectives were: (1) to establish a comprehensive behavioral baseline by evaluating sex preferences in both intra- and cross-species interactions; and (2) to assess whether the Beagle model exhibits reduced sensitivity to experimenter sex, thereby circumventing a key confound in rodent research. This work provides empirical support for the Beagle as a baseline-stable, low-interference model for neuropsychiatric disorders and lays groundwork for its application in mechanistic research and drug development for conditions such as depression and autism spectrum disorder.

## 2. Materials and Methods

### 2.1. Animals

A total of 34 healthy Beagle dogs (17 males and 17 females, aged 8–14 months, body weight 10–18 kg) were obtained from Guangdong National Beagles Resources Research Center (Guangzhou, China), the only state-level platform for canine laboratory animal resources approved by the Ministry of Science and Technology. Three months before behavioral testing, dogs were individually housed at the Kunming Institute of Zoology, Chinese Academy of Sciences in standard cages (1 × 0.8 × 1 m, length × width × height), allowing them to adapt and reducing the influence of social rank, under controlled environmental conditions: a 12 h/12 h dark/light cycle (lights on at 07:00), temperature 16–24 °C, relative humidity 40–70%. Food and water were provided twice daily (08:00–10:00 and 15:00–17:00). All dogs had visual, auditory, and olfactory contact with neighboring conspecifics. All animals were socially housed with conspecifics and had regular contact with caretakers of both sexes during routine husbandry. Female dogs were confirmed by veterinary examination to be in non-estrus throughout the experimental period. All animals were vaccinated and dewormed before the study, and health status was monitored daily by veterinary staff. Allocation of dogs to experimental conditions was performed using a completely randomized design. For dog–dog tests, each of the 34 experimental dogs was randomly assigned to first encounter either a same-sex or opposite-sex unfamiliar control dog, with order counterbalanced across subjects. For human–dog tests, the order of exposure to male versus female experimenters was also randomly determined and balanced. All control dogs (n = 6; 3 males, 3 females) were selected a priori based on their consistent social activity in pre-screening tests and were not used as experimental subjects, and all the control dogs were healthy and relatively sociable.

Sample size was based on previous canine behavioral studies [37]; n = 17 per sex provides 80% power to detect large effects (Cohen’s d > 0.8) at α = 0.05. All behavioral tests were performed at similar time of the day (8:00–12:00 or 13:00–18:00). All animal experimental procedures were approved by the Institutional Animal Care and Use Committee (IACUC) of Kunming Institute of Zoology, Chinese Academy of Sciences (Approval ID: ACUC-OE-2023-02-002-1) and conducted in accordance with institutional and international guidelines for the care and use of laboratory animals.

### 2.2. Dog-Dog Social Interaction Test (DDST)

The dog–dog social interaction test assessed intraspecific social preferences for same or opposite-sex conspecifics. Tests were conducted in a standard experimental arena (5 × 5 × 2.5 m, length × width × height) with ceiling-mounted video cameras for continuous behavioral recording. Each experimental dog was placed simultaneously into the arena with an unfamiliar control Beagle of either the same or opposite sex and allowed to interact freely for 10 min. Control dogs (n = 6; 3 males, 3 females) were selected from the same colony based on consistent social activity (defined as initiating interaction with an unfamiliar conspecific for >5 min in pre-screening tests) and were not used as experimental subjects. The order of same-sex and opposite-sex trials was counterbalanced across experimental dogs, with a minimum 48 h interval between trials. No human interference was allowed during the test.

Two primary behavioral metrics were quantified from video recordings by trained observers blind to experimental conditions: (1) frequency of social initiations—the number of times the experimental dog either actively initiated or responded to social contact (sniffing, approaching, body contact, play solicitation); and (2) total duration of social interaction—the cumulative time the experimental dog remained engaged in active social behavior with the control dog. A social interaction event was defined as any affiliative or investigative behavior involving physical contact or close proximity (<0.5 m) with orientation toward the partner. Events were considered ongoing until the dog either terminated the activity or switched to a different behavioral pattern. To ensure data reliability, only behaviors lasting ≥1 s were included in subsequent analysis.

Within each sex-paired group, experimental dogs were randomly assigned to specific control dogs. Each experimental dog underwent two separate test sessions: one with a same-sex control dog and one with an opposite-sex control dog. A sufficient rest period was ensured between the two sessions for both experimental and control dogs, and no dog (experimental or control) was allowed to participate in two consecutive sessions. The order of the two conditions (same-sex first vs. opposite-sex first) was counterbalanced across experimental dogs.

### 2.3. Human-Dog Social Interaction Test (HDST)

The human–dog social interaction test assessed cross-species social preferences for male versus female human experimenters, using a protocol adapted from Tian R et al. [28].

Habituation Phase: To minimize stress and novelty effects, all dogs underwent a 3-day habituation period before testing. On each day, both male and female experimenters (wearing identical clean lab coats and face masks), individually approached each dog’s home cage for 5 min sessions, twice daily (morning and afternoon). Experimenters remained still or moved slowly in front of the cage, allowing dogs to acclimate to their presence, attire, and general scent without forced interaction. This phase aimed to reduce novelty-induced stress, and approach latencies were not formally recorded or reinforced. Testing Phase: On the test day, the dog was first allowed to explore the empty arena for 2 min. A male or female experimenter then entered and stood quietly at a designated starting point. The dog was allowed to interact freely with the experimenter for 4 min. When the dog initiated social contact (sniffing, making physical contact, or seeking proximity to the experimenter), the experimenter immediately responded by gently stroking the dog’s head and neck, providing positive reinforcement. The order of male and female experimenter trials was counterbalanced across dogs, with a 48 h interval between trials. The duration and frequency of social interactions with the experimenter were quantified from video recordings. All coding was performed by observers blind to experimental conditions. The ages of both male and female experimenters ranged between 20 and 35 years. During the testing period, all experimenters were prohibited from using perfume, smoking, or carrying any noticeable body odor.

### 2.4. Hormone Assays

Blood samples were collected from dogs during the week preceding behavioral testing, between 09:00 and 12:00 to control for circadian variation. Only baseline samples were obtained, and no post-interaction blood samples were collected, because the acute stress of capture and venipuncture would have confounded any endocrine changes induced by the social interaction itself. Dogs were fasted overnight prior to sampling. Venous blood was collected into heparinized tubes, immediately placed on ice, and centrifuged at 3000 rpm for 15 min at 4 °C, and the supernatant was collected. The plasma was aliquoted into 1.5 mL centrifuge tubes, flash-frozen in liquid nitrogen for 15 min and then stored at −80 °C until assay. Plasma concentrations of corticosterone, 5-HT, and dopamine were measured using ELISA kits (Jiangsu Meimian Industrial Co., Ltd., Yancheng, China). According to the manufacturer’s protocols, all samples were run in duplicate. Intra- and inter-assay coefficients of variation were less than 15% for all assays.

### 2.5. Statistical Analysis

All statistics were generated using GraphPad Prism 8.0 software (GraphPad Software, San Diego, CA, USA, www.graphpad.com). Data are presented as mean ± SEM. All data were tested for normality using the Shapiro–Wilk test prior to analysis. For data that met the normality assumption, parametric tests were applied, and group comparisons were performed using unpaired two tailed *t*-tests; for data that did not meet the normality assumption, group comparisons were performed using two-tailed Mann–Whitney U tests. For hormone data, sex differences were analyzed using unpaired *t*-tests. To control the family-wise error rate across eight pair-wise comparisons, a Bonferroni correction was applied, with statistical significance accepted at *p* < 0.00625. Data were tested for normality prior to analysis. Statistical significance was set at *p* < 0.05 (* *p* < 0.05; ** *p* < 0.01, *** *p* < 0.001, **** *p* < 0.0001).

To explore the potential associations between plasma hormone levels and social behavior, Pearson correlation analysis (for normally distributed data) or Spearman rank correlation analysis (for non-normally distributed data) was performed separately for male (n = 15) and female (n = 15) Beagle dogs. Correlations were assessed between baseline plasma concentrations of corticosterone, 5-HT, dopamine and social behavior metrics, including the frequency of social initiations and the total duration of social interaction. All statistical analyses were conducted using GraphPad Prism 8.0 software, with a significance threshold set at *p* < 0.05.

## 3. Results

### 3.1. No Sex-Biased Social Preference in Dog-Dog Interactions

**Figure 1:**
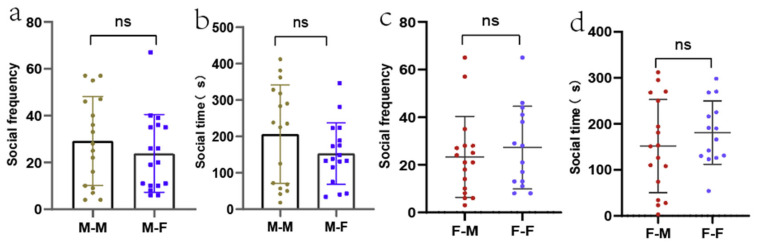
Social behavior in the dog–dog interaction test. (**a**) Frequency of social initiations by male dogs toward male and female conspecifics (n = 17). (**b**) Total interaction time of male dogs with male and female conspecifics. (**c**) Number of social interactions between experimental female dogs (n = 17) and Control male, female dogs and female dogs (n = 14). (**d**) Social interaction time between experimental male Beagle dogs (n = 17) and Control male, female dogs and female dogs (n = 14). Data are presented as mean ± SEM. Tukey’s post hoc test, ns, not significant, M: Male dog, F: Female dog.

For male dogs, the frequency of social initiations did not differ between interactions with male versus female conspecifics. In the dog–dog social interaction test, male dogs showed no significant difference in the frequency of social initiations toward male versus female conspecifics (male partner: 29.18 ± 18.99; female partner: 23.82 ± 16.58; t = 0.8754, df = 32, *p* = 0.3879; Figure 1a). Total interaction time for male dogs also did not differ between male and female partners (male partner: 206.1 ± 135.2 s; female partner: 153.1 ± 84.35 s; t = 1.373, df = 32, *p* = 0.1793; Figure 1b). For female dogs, the frequency of social initiations did not differ between same and opposite-sex partners (female partner: 27.29 ± 17.37; male partner: 23.29 ± 16.99; t = 0.6445, df = 29, *p* = 0.5243; Figure 1c). Total interaction time for female dogs also showed no difference between same- and opposite-sex partners (female partner: 180.7 ± 69 s; male partner: 151.8 ± 101.3 s; t = 0.9084, df = 29, *p* = 0.3712; Figure 1d).

### 3.2. No Experimenter Gender Effect in Human-Dog Interactions

**Figure 2:**
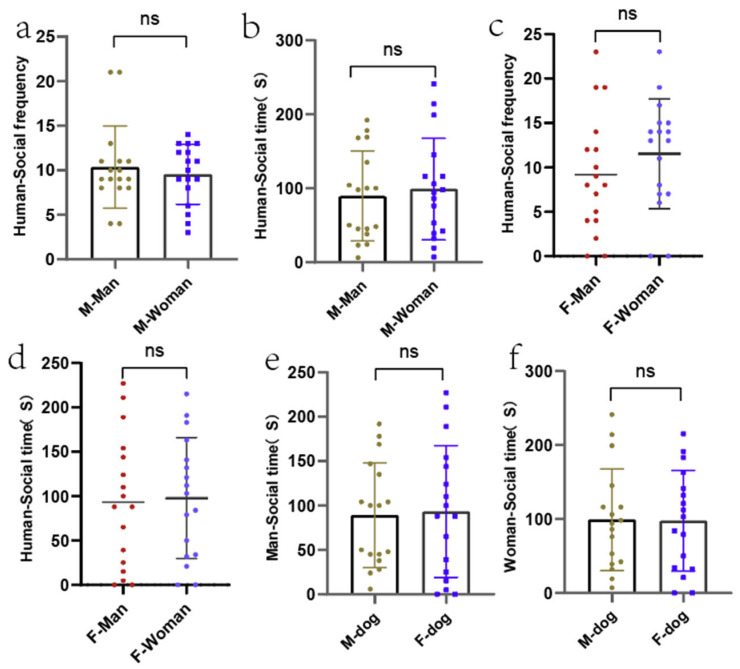
Social behavior in the human–dog interaction test. (**a**,**b**) Male dogs (n = 17) interacting with male and female experimenters: (**a**) number of social interactions, (**b**) interaction time. (**c**,**d**) Female dogs (n = 17) interacting with male and female experimenters: (**c**) number of social interactions, (**d**) interaction time. (**e**,**f**) Comparison of interaction time between male and female dogs (n = 17 each) with (**e**) male experimenter and (**f**) female experimenter. Data are presented as mean ± SEM. Tukey’ s post hoc test, ns, not significant. M: male dog, F: female dog.

In the human–dog social interaction test, male dogs showed no significant difference in the frequency of social initiations toward male versus female experimenters (male experimenter: 10.35 ± 4.609. female experimenter: 9.529 ± 3.375. t(32) = 0.5944, *p* = 0.5564, Figure 2a). Total interaction time for male dogs also did not differ between male and female experimenters (Figure 2b, male experimenter: 89.59 ± 60.71. female experimenter: 99 ± 68.54. t(32) = 0.4238, *p* = 0.6745). For female dogs, the frequency of social initiations did not differ toward male versus female experimenters (Figure 2c, F-Man: 9.17 ± 66.7. F-Woman: 11.53 ± 6.196. t(32) = 1.061, *p* = 0.2965). Total interaction time for female dogs also showed no difference between male and female experimenters (Figure 2d, F-Man: 93.18 ± 74.25 s. F-Woman: 97.71 ± 68 s. t(32) = 0.1855, *p* = 0.8540) toward male versus female experimenters.

We also directly compared male and female dogs’ interaction times with each experimenter gender (Figure 2e,f). When interacting with male experimenters, there was no statistically significant difference in interaction time between male and female dogs (Figure 2e, Male dogs: 89 ± 58.96 s; Female dogs: 93.18 ± 74.25 s, t(32) = 0.1816, *p* = 0.8570). Similarly, no significant difference was found between sexes when interacting with a female experimenter (Figure 2f, Male dogs: 99 ± 68.54 s; Female dogs: 97.71 ± 68 s, t(32) = 0.055, *p* = 0.9536).

### 3.3. Sex Differences in Corticosterone, 5-HT, and Dopamine

**Figure 3:**
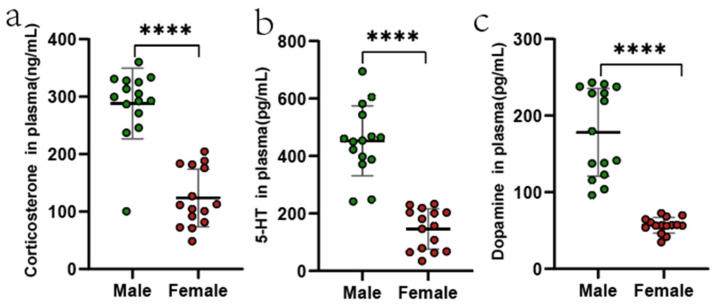
Plasma hormone analysis. (**a**) Plasma corticosterone concentrations. (**b**) Plasma 5-HT concentrations. (**c**) Plasma dopamine concentrations. Plasma samples were collected from male (n = 15) and female (n = 15) Beagle dogs. Data are presented as mean ± SEM, and statistical comparisons were performed using unpaired *t*-tests. Statistical significance was set at *p* < 0.05 (**** *p* < 0.0001).

In contrast to the uniform behavioral results, plasma hormone assays revealed pronounced sex differences in baseline neuroendocrine states (Figure 3). The plasma corticosterone levels were markedly elevated in males compared to female dogs (Males: 288.1 ± 61.5 ng/mL, Female dogs: 123.7 ± 50.28 ng/mL, t(28) = 8.016, *p* < 0.0001, Figure 3a). The plasma 5-HT levels were also significantly higher in males (Male dogs: 452.6 ± 121.8 pg/mL, Female dogs: 145.9 ± 70.13 pg/mL, t(28) = 8.448, *p* < 0.0001). Plasma dopamine levels were significantly higher in male as well (Figure 3c, Male dogs: 178.1 ± 57.28 pg/mL, Female dogs: 56.82 ± 10.22 pg/mL, t(28) = 8.076, *p* < 0.0001).

### 3.4. Lack of Correlation Between Hormone Levels and Social Behavior

**Figure 4:**
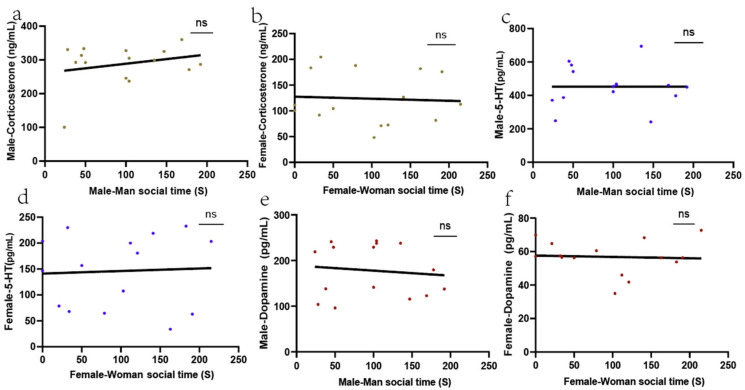
Correlation analysis between social interaction time and plasma hormone concentrations in Beagle dogs. (**a**,**c**,**e**) show correlations for male dogs interacting with male experimenters, with plasma concentrations of corticosterone (**a**), 5-HT (**c**), and dopamine (**e**), respectively. (**b**,**d**,**f**) show correlations for female dogs interacting with female experimenters, with plasma concentrations of corticosterone (**b**), 5-HT (**d**), and dopamine (**f**), respectively. n = 15 per sex. All data were tested for normality prior to analysis, and Pearson correlation analysis was performed. ns, not significant.

Spearman rank correlation revealed no significant correlations between social interaction time and any of the measured hormonal parameters. Interaction time with male experimenters was not correlated with plasma corticosterone in male dogs, (Figure 4a, r = −0.01073, *p* = 0.9719), 5-HT (Figure 4c, r = 0.09123, *p* = 0.7454), or dopamine (Figure 4e, r = −0.2172, *p* = 0.4512) concentrations. Similarly, interaction time with female experimenters showed no significant correlation with plasma corticosterone in female dogs, (Figure 4b, r = −0.05004, *p* = 0.8598), 5-HT (Figure 4d, r =−0.2431, *p* = 0.3797), or dopamine concentrations (Figure 4f, r = −0.2093, *p* = 0.4505). These results suggest that baseline neuroendocrine status may not be linearly associated with social behavior in Beagle dogs under the present testing conditions.

## 4. Discussion

This study investigated sex-biased social preferences in Beagle dogs during intra- and cross-species interactions, and whether baseline neuroendocrine profiles predict such preferences. The primary findings confirmed that Beagles exhibit no sex-biased social preferences toward conspecifics or human experimenters. Notably, despite pronounced sex differences in baseline corticosterone, serotonin, and dopamine (males significantly higher than females), no correlations were found between any hormone and social behavioral measures. Together, these results demonstrate that Beagles show stable and gender-neutral social behavior, and that this behavioral stability is independent of baseline neuroendocrine status.

The absence of experimenter gender effects in Beagles stands in clear contrast to observations in rodent models. In mice and rats, the sex of the human experimenter profoundly influences behavior, physiology, and pharmacological responses. Male experimenter scent reliably induces stress and anxiety-like behaviors and elevates corticosterone [7]. These effects are mediated by olfactory cues that tap into conserved threat circuits, a legacy of the ancestral predator–prey relationship between rodents and humans [8]. The absence of such effects in Beagles points to a fundamental reorganization of social cognition during domestication. Dogs (Canis familiaris) have co-evolved with humans over tens of thousands of years, developing sophisticated socio-cognitive abilities tailored for interspecific communication [34,35]. This domestication process likely selectively enhanced their generalized responsiveness to various human social signals (e.g., gestures, vocal tones) while simultaneously diluting sensitivity to conspecific sex-specific cues. This long history of mutualistic partnership may have shaped a social phenotype that is affiliative rather than threat-reactive [38]. A recent comparison of wolves with and without dog genetic admixture supports this view: admixed wolves showed reduced fear, vigilance, and aggression toward unfamiliar humans compared to non-admixed wolves, suggesting that dog gene introgression attenuates fear-based reactions while preserving general sociability [39].

The dissociation between hormone levels and social behavior represents another key finding. Males had significantly higher baseline corticosterone, serotonin, and dopamine than females, yet in standardized social tests, males and females were indistinguishable, and no correlations emerged between any hormone and any behavioral measure. This dissociation is particularly relevant given that in rodents, corticosterone is closely associated with anxiety-like behaviors [40]. 5-HT and dopamine levels were negatively correlated with social behavior [41,42]. The decoupling observed in Beagles may represent another facet of domestication. Dogs may have evolved more robust social regulatory mechanisms during domestication; selection may have not only reduced fearfulness but also modified the relationship between internal physiological states and behavioral expression. This interpretation is consistent with the broader principle of domestication-driven decoupling. Hansen Wheat et al. demonstrated that suites of correlated domestication-related behaviors have become temporally decoupled in modern dog breeds, showing that selection can disrupt ancestral linkages among behavioral traits [43]. Similarly, Wirobski et al. reported that wolves and dogs show comparable behavioral reactions to social challenges but divergent hormonal profiles [36]. The present results suggest that this decoupling principle may also apply to sex differences in baseline endocrine profiles, extending the phenomenon from cross-species or cross-breed comparisons to within-breed sexual dimorphism. If so, the higher male hormone levels would not reflect a missing brain-behavior link, but rather a domesticated phenotype in which physiological flexibility persists beneath a behaviorally stable surface shared by both sexes.

This behavioral stability extended to both social domains examined. Despite the distinct evolutionary histories of intra- and cross-species interactions, intraspecific behavior rooted in canid pack dynamics and interspecific behavior shaped by coevolution with humans converged on the same outcome: absence of sex-based preferences. This convergence suggests that the observed behavioral stability is not domain-specific but reflects a generalized feature of the domesticated canine social phenotype. In intraspecific contexts, social behavior is modulated by factors beyond sex, such as familiarity and individual relationship history. In cross-species contexts, the absence of experimenter gender effects aligns with evidence that dogs prioritize social intentions and relationships over static attributes such as gender. The differential sensitivity to experimenter sex between rodents and dogs can be further understood by comparing their dominant sensory modalities and cognitive specializations. Rodents primarily rely on olfactory cues for conspecific and heterospecific recognition, making them highly susceptible to human scent as a threat signal [8,12]. In contrast, dogs, as visual-auditory social learners, integrate multimodal information and prioritize cues related to human communicative intent over static features such as gender [23]. This sensory-cognitive shift, driven by domestication, likely contributes to the absence of experimenter gender effects observed in the present study.

These findings have implications for understanding social behavior across species. The experimenter gender effect is a major confound in rodent studies, contributing to poor reproducibility and complicating cross-laboratory comparisons [2,3,4]. The insensitivity to this effect observed in dogs suggests that species with distinct evolutionary histories may require different methodological considerations. More broadly, the decoupling of hormonal state from behavioral output in a domesticated species points to the plasticity of social regulatory mechanisms. This observation may offer insights for research on social behavior, particularly in contexts where separating internal physiological states from behavioral expression is relevant. Disorders involving social dysfunction, such as autism spectrum disorder and depression, often involve complex interactions between neuroendocrine function and social behavior. The present findings suggest that dog models, shaped by domestication to be highly attuned to human social cues while remaining stable across internal states, may provide a complementary perspective to traditional rodent models.

Several limitations should be noted. First, only baseline hormone levels were measured, not stress-induced responses. Thus, we cannot rule out that social interaction might trigger differential hormone changes between sexes, even though baseline levels did not correlate with behavior. We also acknowledge that plasma 5-HT and dopamine reflect peripheral levels and may not directly represent central neurochemistry; they were used here as accessible physiological correlates of the behavioral phenotype, as in prior canine studies [31]. Future studies should incorporate pre- and post-interaction sampling to capture dynamic endocrine responses. Second, the behavioral tests involved only low-stakes, affiliative interactions. More challenging contexts (e.g., exposure to unfamiliar threatening stimuli or resource competition) might reveal sex differences that remain latent under benign conditions. Third, only one breed (Beagle) was studied under standardized laboratory conditions. Whether other dog breeds, or dogs reared in different environments (e.g., pet or shelter dogs), show similar gender-neutral social behavior remains to be tested. Fourth, the sample size (n = 17 per sex) was adequate to detect large effects (Cohen’s d > 0.8) but may have been insufficient to detect small-to-moderate sex differences or weak correlations. Replication in larger cohorts is therefore warranted. Fifth, the experimenters were young adults (20–35 years), and we did not control for the female experimenter’s menstrual cycle. Although no behavioral bias was detected, future studies should systematically assess potential effects of experimenter age and hormonal status. Future research using experimental manipulations (e.g., hormone administration or social conditioning) will be necessary to establish whether hormonal states can influence social behavior in dogs under different conditions. In light of these limitations, future research should address the above points to further test the robustness and generality of the observed patterns.

## 5. Conclusions

In conclusion, the findings suggest that dogs may exhibit stable, gender-neutral social behavior in both intra- and cross-species interactions, despite profound underlying sex differences in baseline corticosterone, 5-HT, and dopamine. The absence of experimenter gender effects may distinguish dogs from rodents and reflects their unique evolutionary history of domestication. The decoupling of hormonal state from behavioral output suggests that social stability may be achieved independently of internal physiological variation, offering a perspective that complements findings from traditional laboratory species.

Taken together, this study provides preliminary evidence that Beagle dogs may serve as a useful translational model with a stable social baseline and reduced susceptibility to experimenter-derived social cues, which could complement rodent models in research on affective and social-cognitive disorders. Independent replication and further experimental studies are needed to confirm and extend these findings.

## Data Availability

The data supporting the findings of this study are included within the article and its Supplementary Materials. Additional information is available from the corresponding author upon reasonable request.

## Supplementary Materials

The following supporting information can be downloaded at https://www.mdpi.com/article/10.3390/ani16111680/s1, Table S1: Social interaction data and plasma hormone concentrations for male and female Beagle dogs.
